# Global Spread of Norovirus GII.17 Kawasaki 308, 2014–2016

**DOI:** 10.3201/eid2308.161138

**Published:** 2017-08

**Authors:** Martin C.W. Chan, Yunwen Hu, Haili Chen, Alexander T. Podkolzin, Ekaterina V. Zaytseva, Jun Komano, Naomi Sakon, Yong Poovorawan, Sompong Vongpunsawad, Thanundorn Thanusuwannasak, Joanne Hewitt, Dawn Croucher, Nikail Collins, Jan Vinjé, Xiaoli L. Pang, Bonita E. Lee, Miranda de Graaf, Janko van Beek, Harry Vennema, Marion P.G. Koopmans, Sandra Niendorf, Mateja Poljsak-Prijatelj, Andrej Steyer, Peter A. White, Jennifer H. Lun, Janet Mans, Tin-Nok Hung, Kirsty Kwok, Kelton Cheung, Nelson Lee, Paul K.S. Chan

**Affiliations:** The Chinese University of Hong Kong, Hong Kong, China (M.C.W. Chan, T.-N. Hung, K. Kwok, K. Cheung, N. Lee, P.K.S. Chan);; Fudan University, Shanghai, China (Y. Hu, H. Chen);; Central Research Institute of Epidemiology, Moscow, Russia (A.T. Podkolzin, E.V. Zaytseva);; Nagoya Medical Center, Nagoya, Japan (J. Komano);; Osaka Prefectural Institute of Public Health, Osaka, Japan (N. Sakon);; Chulalongkorn University, Bangkok, Thailand (Y. Poovorawan, S. Vongpunsawad, T. Thanusuwannasak);; The Institute of Environmental Science and Research, Porirua, New Zealand (J. Hewitt, D. Croucher);; Centers for Disease Control and Prevention, Atlanta, Georgia, USA (N. Collins, J. Vinjé);; University of Alberta, Edmonton, Alberta, Canada (X.L. Pang, B.E. Lee);; Alberta Provincial Laboratory for Public Health, Edmonton, Canada (X.L. Pang); Erasmus MC, Rotterdam, the Netherlands (M. de Graaf, J. van Beek, M.P.G. Koopmans);; National Institute for Public Health and the Environment, Bilthoven, the Netherlands (J. van Beek, H. Vennema, M.P.G. Koopmans);; Robert Koch-Institute, Berlin, Germany (S. Niendorf);; University of Ljubljana, Ljubljana, Slovenia (M. Poljsak-Prijatelj, A. Steyer);; University of New South Wales, Sydney, New South Wales, Australia (P.A. White, J.H. Lun); University of Pretoria, Pretoria, South Africa (J. Mans)

**Keywords:** viruses, basal haplotype, diversification, norovirus, sublineage, transmission, Italy, Romania, Canada, United States, Thailand, the Netherlands, Germany, Slovenia, Australia, New Zealand, China, Hungary, South Korea, Russia, Japan, South Africa, Taiwan, Hong Kong

## Abstract

Analysis of complete capsid sequences of the emerging norovirus GII.17 Kawasaki 308 from 13 countries demonstrated that they originated from a single haplotype since the initial emergence in China in late 2014. Global spread of a sublineage SL2 was identified. A new sublineage SL3 emerged in China in 2016.

Norovirus infections are a leading cause of acute gastroenteritis worldwide in persons of all age groups. Despite the broad genetic diversity, norovirus GII.4 has predominated during the past 20 years (*1*). During winter 2014–15, a new norovirus GII genotype 17 variant, known as Kawasaki 308–like 2014 (GII.17 Kawasaki), emerged and became the predominant genotype in Hong Kong, China (*2*), several major cities of mainland China (*3**,**4*), and Japan (*5*). This variant also was detected sporadically outside of Asia in countries such as Italy, Romania, and the United States (*6**–**8*). This new GII.17 Kawasaki variant is distinct from other GII.17 strains, including the co-circulating Kawasaki 323–like strains; it has 2 characteristic amino acid insertions in the most surface-exposed antigenic region of the major capsid viral protein 1 (VP1) (*2*). These changes have the potential to alter the antigenic properties or the virus–host cell binding preference, raising concern about the global spread of this variant and its replacement of GII.4 variants (*9*). To study the phylodynamic transmission pattern of norovirus GII.17 Kawasaki, we analyzed full-length VP1 nucleotide sequences collected worldwide during late 2014 through early 2016.

## The Study

We chose the region VP1 to analyze because it contained the most hypervariable protruding domain 2 across the norovirus genome and represented most sequences deposited in the public domain. The entire dataset comprised 254 complete VP1 sequences from 13 countries, and all were obtained from samples collected during September 2014–March 2016 (Table). Among them, 129 sequences from 10 countries were determined for this study (Technical Appendix), and the remaining 125 sequences were retrieved from GenBank. These sequences were collected from diverse settings, including outbreaks in healthcare facilities and food-serving sites, sporadic community cases, and hospitalized patients (Technical Appendix Table 1).

**Table:** Number of complete viral protein 1 nucleotide sequences of norovirus genogroup II genotype 17 Kawasaki analyzed from September 2014 to March 2016, grouped by country, source of sequence, and time of collection*

Region and country	Source of sequence			Year of collection, quarter									Total
				2014			2015					2016	
	GenBank	This study		Q3	Q4		Q1	Q2	Q3	Q4		Q1	
Asia													
China													
Hong Kong	81	45		1	26		67	12		2		18	126
Shanghai	3	8			2		1	1		2		5	11
Other cities	31				3		28						31
Other countries													
Japan	2	13					8		2			5	15
South Korea	5				2		2	1					5
Thailand		7			1				1	5			7
Oceania: New Zealand		6							2	2		2	6
Europe													
Germany		5						1		3		1	5
Hungary	1									1			1
Italy	1						1						1
The Netherlands		5					1	2		2			5
Russia		25					1	2	8	12		2	25
Slovenia		4						1	2			1	4
North America													
Canada		6							1	2		3	6
United States	1	5			1			1	2			2	6
Total	125	129		1	35		109	21	18	31		39	254

*Blank cells indicate 0.

**Figure 1:**
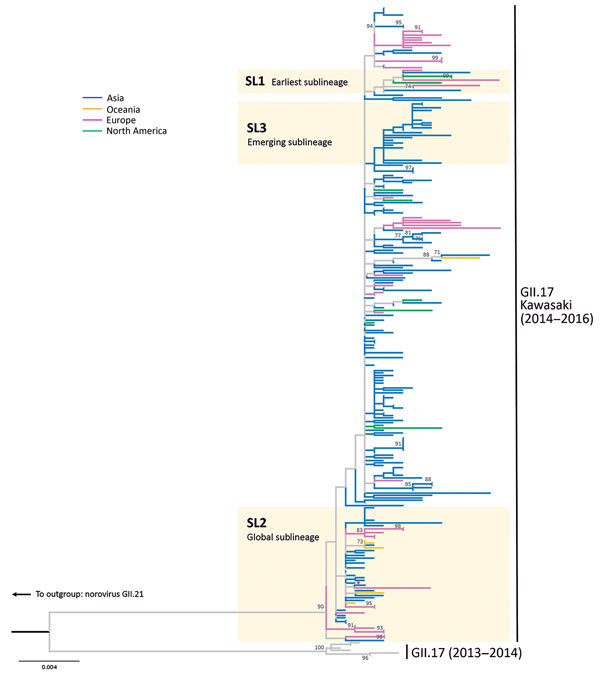
Maximum-likelihood phylogenetic inference of complete viral protein 1 nucleotide sequences of norovirus GII.17 Kawasaki. The tree was constructed using MEGA6 (http://www.megasoftware.net) (Technical Appendix). The red asterisk denotes the reference sequence of GII.17 Kawasaki virus (Hu/GII/JP/2015/GII.P17_GII.17/Kawasaki308; GenBank accession no. LC037415). The tree is rooted to genotype GII.21 (not shown for clarity). Bootstrap values >70 (percentage) are shown at nodes. Sublineages SL1 to SL3 are defined by the topology of haplotype network shown in Figure 2. Branches are colored by the continent of sequence origin. The tree is drawn in scale; scale bar indicates nucleotide substitutions per site.

**Figure 2:**
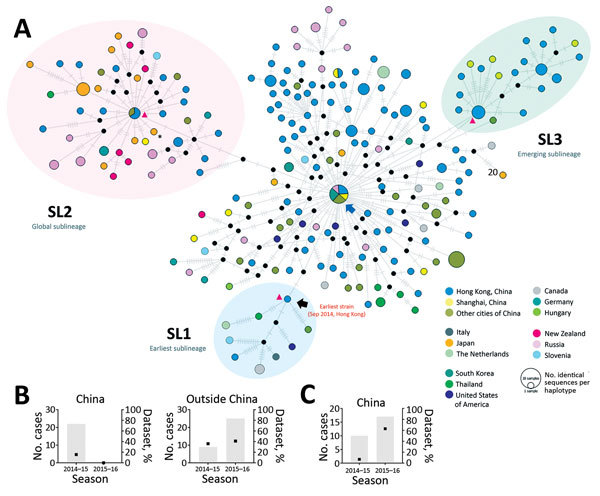
Median-joining haplotype network of 254 complete viral protein 1 nucleotide sequences of norovirus GII.17 Kawasaki. Each vertex represents a unique sampled haplotype. Internal black nodes are unsampled intermediate hypothetical haplotypes. A) Black arrow denotes the first case of norovirus GII.17 Kawasaki in this study (NS-405; collected in September 2014 from Hong Kong). Blue arrow denotes a highly connected basal haplotype from which nearly all haplotypes originated. Vertices are colored by country of collection. Blue shading indicates a sublineage (SL1) genetically closest to the first case of GII.17 Kawasaki virus in this study. Pink shading indicates a sublineage (SL2) with global spread. Green shading indicates an emergent sublineage (SL3) in China in 2016. Vertex size is proportional to the number of sampled sequences sharing the same haplotype. Length of edge is not drawn to scale. Each hatch mark indicates 1 nt difference between connecting haplotypes/nodes. Red triangles represent reference strains of corresponding sublineage (Technical Appendix Table 1). The asterisk denotes the reference sequence of GII.17 Kawasaki virus (Hu/GII/JP/2015/GII.P17_GII.17/Kawasaki308; GenBank accession no. LC037415). Bar charts show the number (gray bars) and percentage (black squares) of cases of sublineages SL2 and SL3 by country in the seasons of 2014–15 (September 2014–June 2015) and 2015–16 (July 2015–March 2016).

GII.17 Kawasaki viruses were found in 13 countries across 4 continents: Canada, China, Germany, Hungary, Italy, Japan, the Netherlands, New Zealand, Russia, Slovenia, South Korea, Thailand, and the United States. Australia and South Africa reported no GII.17 Kawasaki as of mid-2015 and early 2016, respectively. Maximum-likelihood phylogenetic inference showed different genetic clusters within GII.17 Kawasaki, indicating rapid genetic diversification of viral population during spread (Figure 1; Technical Appendix Figure). Sequences from the same continent scattered into different genetic clusters, inferring multiple introduction and frequent transmission events. To investigate the virus transmission pattern, we constructed a median-joining haplotype network based on complete VP1 nucleotide sequences (Technical Appendix). Overall, the 254 VP1 sequences comprised 207 different haplotypes (Figure 2). We identified a highly connected basal haplotype (Figure 2) that consisted of 8 identical VP1 sequences collected in the initial phase of the epidemic during November 2014–March 2015 from 6 cities mostly in Asia (2 from Hong Kong; 1 from Shanghai, China; 1 from Guangzhou, China; 1 from Taiwan; 2 from South Korea; and 1 from Russia). The same basal haplotype was concluded using integer neighbor-joining and tight span walker network models. The central node might represent a competent virus haplotype capable of replicating and spreading efficiently among humans and from which nearly all haplotypes originated. We found only 2 nucleotide differences without amino acid change between the basal haplotype and the first case-patient with GII.17 Kawasaki virus in this study (NS-405; collected in September 2014 from Hong Kong) (Figure 2, black arrow). We determined complete genomes that comprised the basal haplotype for this study for the 2 Hong Kong strains and downloaded data for the 2 South Korea strains. These viruses had 4 unique amino acid substitutions distinct from NS-405: 2 in the nonstructural polyprotein (A187D in N terminal protein and N739S in protease) and 2 in VP2 (K58R and A89S; outside of the VP1-interacting domain) (Technical Appendix Table 2). Substitution in the protease might mediate changes in the cleavage efficiency of the polyprotein in norovirus replication (*10*). Although we noted no substitutions in the RNA-dependent RNA polymerase, N terminal protein and VP2 were previously implicated in modulating polymerase activity, virus tropism, and persistence (*11**,**12*). The 4 non-VP1 residues may affect viral fitness of the emergent GII.17 Kawasaki in humans; however, functional characterization is required (*13*).

We identified 3 important sublineages by topology (Figure 2). Viruses belonging to sublineage SL1 (Figure 2, blue shading) clustered closest to the first GII.17 Kawasaki isolate in this study. SL1 included strains from 6 countries outside of China across 3 continents: Thailand (collected in October 2014), United States (November 2014), Italy and the Netherlands (February 2015), Slovenia (August 2015), and Canada (December 2015–January 2016). The global spread of GII.17 Kawasaki viruses within a few months after the initial emergence in China in late 2014 highlights rapid transmissibility of these viruses. Despite the molecular evidence of early global presence of SL1, the apparent limited circulation of this sublineage is intriguing. SL1 was the only sublineage not originating from the basal haplotype but directly from the earliest NS-405. Sequence analysis of the other 2 SL1 complete genomes available, collected from the United States (Hu/GII.17/Gaithersburg/2014/U.S.; GenBank accession no. KR083017) and Taiwan (Hu/GII.17/CGMH70/2015/TW; GenBank accession no. KR154231), found none of the 4 non-VP1 substitutions observed in the basal haplotype. In this dataset, viruses belonging to sublineage SL2 had the most cases and widest geographic breadth (Figure 2, pink shading). SL2 was detected in 6 countries outside of China across 3 continents (Germany, Japan, New Zealand, Russia, Slovenia, and Thailand) and most of the non-China sequences from 2014–15 (36%) and 2015–16 (41%) seasons belonged to this sublineage (Figure 2, inset). The most successful SL2 might have an advantage to global spread, although we cannot rule out sampling bias. During the 2015–16 season, SL2 continued to circulate over a wide geographic area, although none of the sequences from China belonged to this sublineage. Instead, sublineage SL3, first detected in January 2015 as a minority (7%) in China in the 2014–15 season, became the predominant (63%) circulating GII.17 Kawasaki virus in both southern (Hong Kong) and eastern (Shanghai) parts of China during 2015–16 among sequences analyzed (Figure 2, green shading and inset). No sequences from other countries clustered into SL3. This emerging sublineage highlights that GII.17 Kawasaki viruses were still circulating and, more important, rapidly evolving in various regions of China. Robustness of sublineage topology was confirmed in the phylogenetic tree (Figure 1).

## Conclusions

We determined the complete VP1 sequences of 129 GII.17 Kawasaki strains from 10 countries. Our analyses suggest that the new GII.17 Kawasaki originated from a single haplotype from which rapid genetic diversification into multiple sublineages occurred during global spread after the initial emergence in China in late 2014. Norovirus diversification into sublineages provides a preepidemic virus pool from which new pandemic GII.4 variants emerged (*14*). Although our study is limited by its focus on VP1 sequence analysis and not on virus genomes, it nevertheless is a good demonstration that a global network of norovirus laboratories sharing virus sequence information can delineate virus transmission pattern upon spread.

Technical Appendix Complete norovirus viral protein 1 (VP1) gene sequencing and phylogenetic and haplotype network analyses; list of complete VP1 nucleotide sequences of norovirus GII.17 Kawasaki used in median-joining haplotype network analysis; genomewide identification of 4-aa substitutions that can affect viral fitness of norovirus GII.17 Kawasaki in humans; and maximum-likelihood phylogenetic inference of complete VP1 nucleotide sequences of norovirus GII.17 Kawasaki.
