# Supplementary material for: Global Spread of Norovirus GII.17 Kawasaki 308, 2014–2016
Source: Emerg Infect Dis. 2017 Aug;23(8):1350–4. doi: 10.3201/eid2308.161138 (PMC5547775; doi:10.3201/eid2308.161138)
Supplement: Technical Appendix — Complete norovirus viral protein 1 (VP1) gene sequencing and phylogenetic and haplotype network analyses; list of complete VP1 nucleotide sequences of norovirus GII.17 Kawasaki used in median-joining haplotype network analysis; genomewide identification of 4-aa substitutions that can affect viral fitness of norovirus GII.17 Kawasaki in humans; and maximum-likelihood phylogenetic inference of complete VP1 nucleotide sequences of norovirus GII.17 Kawasaki. [file 16-1138-Techapp-s1.pdf]

Article DOI: https://doi.org/10.3201/eid2308.161138

# Global Spread of Norovirus GII.G17 Kawasaki 308, 2014–2016

## Technical Appendix

## Complete Norovirus VP1 Gene Sequencing

Five collaborating sites (Canada, Germany, Hong Kong, New Zealand, and Russia) determined complete viral protein 1 (VP1) gene sequences by first generating ≈2.7-kb amplicons covering 3′ end of virus genomes using high-fidelity DNA polymerase, followed by Sanger sequencing as previously described (*1*). Remaining sites used their own in-house protocols for sequencing.

## Phylogenetic Analysis

Phylogenetic inference was performed using the maximum-likelihood method. Best substitution matrix, Kimura 2-parameter, was determined in MEGA6 (http://www.megasoftware.net). A gamma distribution of rate variation that enabled invariable sites was used. The tree with the highest log likelihood is shown. Tree confidence was assessed by bootstrapping with 1,000 iterations. All nucleotide positions with gaps and missing data were excluded. The final dataset contained 1,603 positions for analysis. The tree was rooted to an outgroup (norovirus GII.21; strain GII.21/IF1998/IQ/2003; GenBank accession no. AY675554).

## Haplotype Network Analysis

Haplotype network analysis has been widely used to study virus transmission pattern, such as the Ebola virus (*2*,*3*), and it works best on closely related strains. A median-joining haplotype network was constructed using PopART version 1.7 (http://popart.otago.ac.nz) based on complete VP1 nucleotide sequences of norovirus GII.17 Kawasaki strains collected during 2014–2016. Kawasaki323-like strains (i.e., those collected before mid-2014) were excluded

because they were genetically distant from Kawasaki308-like strains that circulated predominantly in late 2014 onward and thus were not suitable for haplotype network analysis.

## References


1. Chan MC, Lee N, Hung TN, Kwok K, Cheung K, Tin EK, et al. Rapid emergence and predominance of a broadly recognizing and fast-evolving norovirus GII.17 variant in late 2014. Nat Commun. 2015;6:10061. PubMed http://dx.doi.org/10.1038/ncomms10061

2. Ladner JT, Wiley MR, Mate S, Dudas G, Prieto K, Lovett S, et al. Evolution and spread of Ebola virus in Liberia, 2014–2015. Cell Host Microbe. 2015;18:659–69. PubMed http://dx.doi.org/10.1016/j.chom.2015.11.008

3. Mate SE, Kugelman JR, Nyenswah TG, Ladner JT, Wiley MR, Cordier-Lassalle T, et al. Molecular evidence of sexual transmission of Ebola virus. N Engl J Med. 2015;373:2448–54. PubMed http://dx.doi.org/10.1056/NEJMoa1509773


**Technical Appendix Table 1.** List of 254 complete viral protein 1 nucleotide sequences of norovirus genogroup II genotype 17 (GII.17) Kawasaki used in median-joining haplotype network analysis*

| Counter | Strain Name | GenBank Accession no. | Year and month of collection | City/Province/Country | Remarks |
|---|---|---|---|---|---|
| 1 | NS-405 | KP902566 | 2014 Sep | Hong Kong | Sublineage SL1 reference |
| 2 | NS-438 | KP902567 | 2014 Nov | Hong Kong | |
| 3 | NS-455 | KP902568 | 2014 Nov | Hong Kong | |
| 4 | NS-456 | KP902569 | 2014 Nov | Hong Kong | |
| 5 | NS-463 | KP902570 | 2014 Dec | Hong Kong | |
| 6 | NS-469 | KT315668 | 2014 Dec | Hong Kong | |
| 7 | NS-471 | KU561224 | 2014 Dec | Hong Kong | This study |
| 8 | NS-472 | KT315669 | 2014 Dec | Hong Kong | |
| 9 | NS-475 | KT315670 | 2014 Dec | Hong Kong | |
| 10 | NS-476 | KT315671 | 2014 Dec | Hong Kong | |
| 11 | NS-477 | KU561225 | 2014 Dec | Hong Kong | This study |
| 12 | NS-478 | KT315672 | 2014 Dec | Hong Kong | |
| 13 | NS-480 | KP902571 | 2014 Dec | Hong Kong | |
| 14 | NS-481 | KU561226 | 2014 Dec | Hong Kong | This study |
| 15 | NS-482 | KP902572 | 2014 Dec | Hong Kong | |
| 16 | NS-483 | KP902573 | 2014 Dec | Hong Kong | |
| 17 | NS-484 | KU561227 | 2014 Dec | Hong Kong | This study |
| 18 | NS-486 | KT315673 | 2014 Dec | Hong Kong | |
| 19 | NS-488 | KU561228 | 2014 Dec | Hong Kong | This study |
| 20 | NS-491 | KP698928 | 2014 Dec | Hong Kong | |
| 21 | NS-492 | KP902574 | 2014 Dec | Hong Kong | |
| 22 | NS-493 | KP902575 | 2014 Dec | Hong Kong | |
| 23 | NS-494 | KP698929 | 2014 Dec | Hong Kong | |
| 24 | NS-498 | KU561229 | 2014 Dec | Hong Kong | This study |
| 25 | NS-500 | KP902576 | 2014 Dec | Hong Kong | |
| 26 | NS-502 | KP902577 | 2014 Dec | Hong Kong | |
| 27 | NS-503 | KP902578 | 2014 Dec | Hong Kong | |
| 28 | NS-504 | KU561230 | 2015 Jan | Hong Kong | This study |
| 29 | NS-506 | KP902579 | 2015 Jan | Hong Kong | |
| 30 | NS-511 | KP698930 | 2015 Jan | Hong Kong | |
| 31 | NS-512 | KP902580 | 2015 Jan | Hong Kong | |
| 32 | NS-513 | KP698931 | 2015 Jan | Hong Kong | |
| 33 | NS-514 | KP902581 | 2015 Jan | Hong Kong | |
| 34 | NS-517 | KP902582 | 2015 Jan | Hong Kong | Sublineage SL2 reference |
| 35 | NS-520 | KP902583 | 2015 Jan | Hong Kong | |
| 36 | NS-521 | KP902584 | 2015 Jan | Hong Kong | |

| Counter | Strain Name | GenBank Accession no. | Year and month of collection | City/Province/Country | Remarks |
|---|---|---|---|---|---|
| 37 | NS-522 | KT315674 | 2015 Jan | Hong Kong | |
| 38 | NS-523 | KT315675 | 2015 Jan | Hong Kong | |
| 39 | NS-528 | KP902585 | 2015 Jan | Hong Kong | |
| 40 | NS-533 | KT315676 | 2015 Jan | Hong Kong | |
| 41 | NS-534 | KT315677 | 2015 Jan | Hong Kong | |
| 42 | NS-535 | KU561231 | 2015 Jan | Hong Kong | This study |
| 43 | NS-536 | KU561232 | 2015 Jan | Hong Kong | This study |
| 44 | NS-537 | KT315678 | 2015 Jan | Hong Kong | |
| 45 | NS-539 | KT315679 | 2015 Jan | Hong Kong | |
| 46 | NS-541 | KU561233 | 2015 Jan | Hong Kong | This study |
| 47 | NS-543 | KU561234 | 2015 Jan | Hong Kong | This study |
| 48 | NS-546 | KU561235 | 2015 Jan | Hong Kong | This study |
| 49 | NS-548 | KU561236 | 2015 Jan | Hong Kong | This study |
| 50 | NS-549 | KP902586 | 2015 Jan | Hong Kong | |
| 51 | NS-556 | KP902587 | 2015 Jan | Hong Kong | |
| 52 | NS-560 | KT315680 | 2015 Jan | Hong Kong | |
| 53 | NS-565 | KP902588 | 2015 Jan | Hong Kong | |
| 54 | NS-570 | KT315681 | 2015 Jan | Hong Kong | |
| 55 | NS-574 | KP902589 | 2015 Jan | Hong Kong | |
| 56 | NS-575 | KP902590 | 2015 Jan | Hong Kong | |
| 57 | NS-576 | KU561237 | 2015 Jan | Hong Kong | This study |
| 58 | NS-579 | KT315682 | 2015 Feb | Hong Kong | |
| 59 | NS-580 | KU561238 | 2015 Feb | Hong Kong | This study |
| 60 | NS-582 | KT315683 | 2015 Feb | Hong Kong | |
| 61 | NS-583 | KU561239 | 2015 Feb | Hong Kong | This study |
| 62 | NS-586 | KT315684 | 2015 Feb | Hong Kong | |
| 63 | NS-589 | KT315685 | 2015 Feb | Hong Kong | |
| 64 | NS-592 | KT315686 | 2015 Feb | Hong Kong | |
| 65 | NS-593 | KT315687 | 2015 Feb | Hong Kong | |
| 66 | NS-594 | KU561240 | 2015 Feb | Hong Kong | This study |
| 67 | NS-595 | KU561241 | 2015 Feb | Hong Kong | This study |
| 68 | NS-599 | KT315688 | 2015 Feb | Hong Kong | |
| 69 | NS-600 | KT315689 | 2015 Feb | Hong Kong | |
| 70 | NS-602 | KT315690 | 2015 Feb | Hong Kong | |
| 71 | NS-603 | KT315691 | 2015 Feb | Hong Kong | |
| 72 | NS-604 | KT315692 | 2015 Feb | Hong Kong | |
| 73 | NS-606 | KT315693 | 2015 Feb | Hong Kong | |
| 74 | NS-610 | KU561242 | 2015 Feb | Hong Kong | This study |
| 75 | NS-611 | KT315694 | 2015 Feb | Hong Kong | |
| 76 | NS-612 | KT315695 | 2015 Mar | Hong Kong | Sublineage SL3 reference |
| 77 | NS-613 | KU561248 | 2015 Mar | Hong Kong | This study; basal haplotype |
| 78 | NS-614 | KU561243 | 2015 Mar | Hong Kong | This study |
| 79 | NS-616 | KT315696 | 2015 Mar | Hong Kong | Basal haplotype |
| 80 | NS-619 | KT315697 | 2015 Mar | Hong Kong | |
| 81 | NS-622 | KU561244 | 2015 Mar | Hong Kong | This study |
| 82 | NS-627 | KT315698 | 2015 Mar | Hong Kong | |
| 83 | NS-629 | KT315699 | 2015 Mar | Hong Kong | |
| 84 | NS-634 | KT315700 | 2015 Mar | Hong Kong | |
| 85 | NS-635 | KU561245 | 2015 Mar | Hong Kong | This study |
| 86 | NS-636 | KT315701 | 2015 Mar | Hong Kong | |
| 87 | NS-637 | KT315702 | 2015 Mar | Hong Kong | |
| 88 | NS-639 | KT315703 | 2015 Mar | Hong Kong | |
| 89 | NS-641 | KT315704 | 2015 Mar | Hong Kong | |
| 90 | NS-643 | KT315705 | 2015 Mar | Hong Kong | |
| 91 | NS-647 | KT315706 | 2015 Mar | Hong Kong | |
| 92 | NS-648 | KT315707 | 2015 Mar | Hong Kong | |
| 93 | NS-649 | KT315708 | 2015 Mar | Hong Kong | |
| 94 | NS-650 | KT315709 | 2015 Mar | Hong Kong | |
| 95 | NS-653 | KT315710 | 2015 Apr | Hong Kong | |
| 96 | NS-655 | KT315711 | 2015 Apr | Hong Kong | |
| 97 | NS-656 | KT315712 | 2015 Apr | Hong Kong | |
| 98 | NS-657 | KT315713 | 2015 Apr | Hong Kong | |
| 99 | NS-658 | KT315714 | 2015 Apr | Hong Kong | |
| 100 | NS-659 | KT315715 | 2015 Apr | Hong Kong | |
| 101 | NS-662 | KT315716 | 2015 Apr | Hong Kong | |
| 102 | NS-667 | KT315717 | 2015 Apr | Hong Kong | |
| 103 | NS-670 | KT315718 | 2015 May | Hong Kong | |
| 104 | NS-671 | KT315719 | 2015 May | Hong Kong | |

| Counter | Strain Name | GenBank Accession no. | Year and month of collection | City/Province/Country | Remarks |
|---|---|---|---|---|---|
| 105 | NS-679 | KU561246 | 2015 Jun | Hong Kong | This study |
| 106 | NS-680 | KU561247 | 2015 Jun | Hong Kong | This study |
| 107 | NS-767 | KX168437 | 2015 Oct | Hong Kong | This study |
| 108 | NS-861 | KX168438 | 2015 Dec | Hong Kong | This study |
| 109 | NS-863 | KX168439 | 2016 Jan | Hong Kong | This study |
| 110 | NS-866 | KX168440 | 2016 Jan | Hong Kong | This study |
| 111 | NS-880 | KX168441 | 2016 Jan | Hong Kong | This study |
| 112 | NS-882 | KX168442 | 2016 Jan | Hong Kong | This study |
| 113 | NS-892 | KX168443 | 2016 Feb | Hong Kong | This study |
| 114 | NS-896 | KX168444 | 2016 Feb | Hong Kong | This study |
| 115 | NS-899 | KX168445 | 2016 Feb | Hong Kong | This study |
| 116 | NS-901 | KX168446 | 2016 Feb | Hong Kong | This study |
| 117 | NS-907 | KX168447 | 2016 Feb | Hong Kong | This study |
| 118 | NS-911 | KX168448 | 2016 Feb | Hong Kong | This study |
| 119 | NS-917 | KX168449 | 2016 Mar | Hong Kong | This study; sublineage SL1 reference |
| 120 | NS-920 | KX168450 | 2016 Mar | Hong Kong | This study |
| 121 | NS-922 | KX168451 | 2016 Mar | Hong Kong | This study |
| 122 | NS-928 | KX168452 | 2016 Mar | Hong Kong | This study |
| 123 | NS-930 | KX168453 | 2016 Mar | Hong Kong | This study |
| 124 | NS-935 | KX168454 | 2016 Mar | Hong Kong | This study |
| 125 | NS-936 | KX168455 | 2016 Mar | Hong Kong | This study |
| 126 | NS-942 | KX168456 | 2016 Mar | Hong Kong | This study |
| 127 | 41621 | KR020503 | 2014 Dec | Guangzhou | |
| 128 | GZ2014-L311 | KT149168 | 2014 Dec | Guangzhou | |
| 129 | GZ2014-L313 | KT149169 | 2014 Dec | Guangzhou | |
| 130 | GZ2015-L324 | KT149170 | 2015 Jan | Guangzhou | |
| 131 | GZ2015-L325 | KT149171 | 2015 Jan | Guangzhou | |
| 132 | GZ2015-L337 | KT149172 | 2015 Jan | Guangzhou | |
| 133 | GZ2015-L339 | KT149173 | 2015 Jan | Guangzhou | |
| 134 | GZ2015-L340 | KT149174 | 2015 Jan | Guangzhou | |
| 135 | GZ2015-L343 | KT149175 | 2015 Jan | Guangzhou | |
| 136 | GZ2015-L362 | KT149176 | 2015 Mar | Guangzhou | |
| 137 | JSSZ14313 | KR270442 | 2015 Mar | Jiangsu | |
| 138 | JSZJ14003 | KR270443 | 2015 Mar | Jiangsu | |
| 139 | JSCZ14010 | KR270444 | 2015 Mar | Jiangsu | |
| 140 | JSWX14012 | KR270445 | 2015 Mar | Jiangsu | |
| 141 | JSSZ15080 | KR270446 | 2015 Mar | Jiangsu | |
| 142 | JSXZ15035 | KR270447 | 2015 Mar | Jiangsu | |
| 143 | JSWX15027 | KR270448 | 2015 Mar | Jiangsu | |
| 144 | JSNT15033 | KR270449 | 2015 Mar | Jiangsu | |
| 145 | Kawasaki308 | LC037415 | 2015 Feb | Japan | |
| 146 | HN01 | KT992785 | 2015 Mar | Nanyang | |
| 147 | HN02 | KT992786 | 2015 Mar | Nanyang | |
| 148 | HN03 | KT992787 | 2015 Mar | Nanyang | |
| 149 | HN04 | KT992788 | 2015 Mar | Nanyang | |
| 150 | HN05 | KT992789 | 2015 Mar | Nanyang | |
| 151 | HNkaohao | KT992790 | 2015 Mar | Nanyang | |
| 152 | MIY2 | LC101820 | 2015 Feb | Japan | |
| 153 | 152642 | KP864102 | 2015 Jan | Shanghai | |
| 154 | 142700 | KP864103 | 2014 Nov | Shanghai | |
| 155 | 142661 | KP864104 | 2014 Dec | Shanghai | |
| 156 | CGMH69 | KR154230 | 2015 Jan | Taoyuan, Taiwan | |
| 157 | CGMH70 | KR154231 | 2015 Feb | Taoyuan, Taiwan | |
| 158 | 15-AD-2 | KR052019 | 2015 Feb | Taichung, Taiwan | |
| 159 | 15-AH-1 | KR052020 | 2015 Feb | Changhua, Taiwan | |
| 160 | 15-AP-1 | KR052021 | 2015 Feb | Hsinchu, Taiwan | |
| 161 | 15-R-4 | KR052022 | 2015 Jan | Yunlin, Taiwan | |
| 162 | ZHITHC-12 | KT253245 | 2015 Jan | Zhuhai | |
| 163 | Gaithersburg | KR083017 | 2014 Nov | USA | |
| 164 | PR668 | KT346356 | 2015 Feb | Italy | |
| 165 | CAU-192 | KU561252 | 2014 Nov | South Korea | |
| 166 | CAU-265 | KU561253 | 2014 Dec | South Korea | |
| 167 | CAU-267 | KU561254 | 2015 Jan | South Korea | |
| 168 | CAU-283 | KU561255 | 2015 Mar | South Korea | |
| 169 | CAU-289 | KU561256 | 2015 Apr | South Korea | |
| 170 | HUN5737 | KX024652 | 2015 Oct | Hungary | |
| 171 | 152808 | KU953391 | 2015 May | Shanghai | This study |

| Counter | Strain Name | GenBank Accession no. | Year and month of collection | City/Province/Country | Remarks |
|---|---|---|---|---|---|
| 172 | 1513140 | KU953392 | 2015 Dec | Shanghai | This study |
| 173 | 1513181 | KU953393 | 2015 Dec | Shanghai | This study |
| 174 | 1613155 | KU953394 | 2016 Jan | Shanghai | This study |
| 175 | 1613179 | KU953395 | 2016 Jan | Shanghai | This study |
| 176 | 1613225 | KU953396 | 2016 Feb | Shanghai | This study |
| 177 | 1613305 | KU953397 | 2016 Feb | Shanghai | This study |
| 178 | 1613306 | KU953398 | 2016 Feb | Shanghai | This study |
| 179 | AlbertaEI331 | KX171414 | 2015 Aug | Canada | This study |
| 180 | 11 | KX420891 | 2015 Dec | Canada | This study |
| 181 | 12 | KX420892 | 2015 Dec | Canada | This study |
| 182 | 13 | KX420893 | 2016 Jan | Canada | This study |
| 183 | 14 | KX420894 | 2016 Feb | Canada | This study |
| 184 | 15 | KX420895 | 2016 Feb | Canada | This study |
| 185 | 22478 | KX216782 | 2015 Mar | Russia | This study |
| 186 | 22692 | KX216784 | 2015 May | Russia | This study |
| 187 | 22706 | KX216787 | 2015 Jun | Russia | This study |
| 188 | 22833 | KX216783 | 2015 Jul | Russia | This study |
| 189 | 22852 | KX216786 | 2015 Jul | Russia | This study |
| 190 | 22962 | KX216794 | 2015 Jul | Russia | This study |
| 191 | 23079 | KX216785 | 2015 Aug | Russia | This study |
| 192 | 23108 | KX216795 | 2015 Sep | Russia | This study |
| 193 | 23110 | KX216796 | 2015 Sep | Russia | This study |
| 194 | 23123 | KX216789 | 2015 Sep | Russia | This study |
| 195 | 23233 | KX216793 | 2015 Sep | Russia | This study |
| 196 | 23249 | KX216798 | 2015 Oct | Russia | This study |
| 197 | 23251 | KX216799 | 2015 Oct | Russia | This study |
| 198 | 23289 | KX216806 | 2015 Oct | Russia | This study |
| 199 | 23308 | KX216797 | 2015 Nov | Russia | This study |
| 200 | 23376 | KX216800 | 2015 Nov | Russia | This study |
| 201 | 23377 | KX216801 | 2015 Nov | Russia | This study |
| 202 | 23378 | KX216792 | 2015 Dec | Russia | This study |
| 203 | 23382 | KX216802 | 2015 Dec | Russia | This study |
| 204 | 23383 | KX216803 | 2015 Dec | Russia | This study |
| 205 | 23392 | KX216788 | 2015 Nov | Russia | This study |
| 206 | 23395 | KX216791 | 2015 Dec | Russia | This study |
| 207 | 23406 | KX216790 | 2015 Dec | Russia | This study |
| 208 | 23438 | KX216804 | 2016 Feb | Russia | This study |
| 209 | 23440 | KX216805 | 2016 Feb | Russia | This study |
| 210 | 14–273 | LC148844 | 2015 Jan | Japan | This study |
| 211 | 14–283 | LC148845 | 2015 Jan | Japan | This study |
| 212 | 14–332 | LC148846 | 2015 Jan | Japan | This study |
| 213 | 14–346 | LC148847 | 2015 Jan | Japan | This study |
| 214 | 14–394 | LC148848 | 2015 Feb | Japan | This study |
| 215 | 14–508 | LC148849 | 2015 Mar | Japan | This study |
| 216 | 15–157 | LC148850 | 2015 Jul | Japan | This study |
| 217 | 15–208 | LC148851 | 2015 Aug | Japan | This study |
| 218 | 15–377 | LC148852 | 2016 Jan | Japan | This study |
| 219 | 15–399 | LC148853 | 2016 Jan | Japan | This study |
| 220 | 15–428 | LC148854 | 2016 Jan | Japan | This study |
| 221 | 15–479 | LC148855 | 2016 Mar | Japan | This study |
| 222 | 15–493 | LC148856 | 2016 Mar | Japan | This study |
| 223 | Ljubljana1662 | KT591501 | 2015 Jun | Slovenia | This study |
| 224 | Ljubljana1758 | KX134669 | 2015 Jul | Slovenia | This study |
| 225 | Ljubljana1962 | KX134670 | 2015 Aug | Slovenia | This study |
| 226 | Ljubljana535 | KX134671 | 2016 Mar | Slovenia | This study |
| 227 | 15-G1181.01 | KX244850 | 2015 Nov | Germany | This study |
| 228 | 15-G1182.01 | KX244851 | 2015 Nov | Germany | This study |
| 229 | 15-G1200.01 | KX244853 | 2015 Oct | Germany | This study |
| 230 | 15-G1269.01 | KX244852 | 2015 Apr | Germany | This study |
| 231 | 16-G0188.01 | KX244854 | 2016 Feb | Germany | This study |
| 232 | Veldhoven219 | KX424646 | 2015 Feb | The Netherlands | This study |
| 233 | Almere278 | KX424647 | 2015 May | The Netherlands | This study |
| 234 | Almere279 | KX424648 | 2015 May | The Netherlands | This study |
| 235 | Heemskerk337 | KX424649 | 2015 Nov | The Netherlands | This study |
| 236 | Heemskerk336 | KX424650 | 2015 Nov | The Netherlands | This study |
| 237 | 3000467356 | MF172092 | 2015 May | USA | This study |
| 238 | 3000467424 | MF172093 | 2015 Jul | USA | This study |
| 239 | 3000467425 | MF172094 | 2015 Jul | USA | This study |

| Counter | Strain Name | GenBank Accession no. | Year and month of collection | City/Province/Country | Remarks |
|---|---|---|---|---|---|
| 240 | 3000508986 | MF172095 | 2016 Jan | USA | This study |
| 241 | 3000509166 | MF172096 | 2016 Jan | USA | This study |
| 242 | B1995 | KX346699 | 2014 Oct | Thailand | This study |
| 243 | B2194 | KX346705 | 2015 Sep | Thailand | This study |
| 244 | B2306 | KX346700 | 2015 Oct | Thailand | This study |
| 245 | B2316 | KX346701 | 2015 Nov | Thailand | This study |
| 246 | B2387 | KX346702 | 2015 Nov | Thailand | This study |
| 247 | B2395 | KX346703 | 2015 Nov | Thailand | This study |
| 248 | B2459 | KX346704 | 2015 Dec | Thailand | This study |
| 249 | 15NV570 | KX371107 | 2015 Sep | New Zealand | This study |
| 250 | 15NV581 | KX371108 | 2015 Sep | New Zealand | This study |
| 251 | 15NV796 | KX371109 | 2015 Nov | New Zealand | This study |
| 252 | 15NV806 | KX371110 | 2015 Nov | New Zealand | This study |
| 253 | 16NV021 | KX371111 | 2016 Jan | New Zealand | This study |
| 254 | 16NV149 | KX371112 | 2016 Jan | New Zealand | This study |

*Red text denotes the first case of norovirus GII.17 Kawasaki in this study; blue text denotes strains comprising the competent virus basal haplotype; and green text denotes reference strains of sublineages SL1, SL2 and SL3. Blank cells indicate that sequences were downloaded from GenBank.

**Technical Appendix Table 2.** Genomewide identification of 4-aa substitutions that can affect viral fitness of norovirus GII.17 Kawasaki in humans*

| Protein/residue position | Reference† | NS-613‡ | NS-616‡ | CAU-192‡ | CAU-267‡ |
|---|---|---|---|---|---|
| Polyprotein, ORF1 | | | | | |
| 53 | T | T | T | **A** | T |
| 164 | L | **F** | L | L | L |
| 187§ | A | **D** | **D** | **D** | **D** |
| 739§ | N | **S** | **S** | **S** | **S** |
| 974 | L | L | **F** | L | L |
| 1194 | G | G | G | G | **S** |
| 1674 | F | F | F | **S** | **S** |
| VP2, ORF3 | | | | | |
| 58§ | K | **R** | **R** | **R** | **R** |
| 89§ | A | **S** | **S** | **S** | **S** |
| 111 | T | T | **A** | **T** | T |
| 136 | K | K | **R** | **K** | K |

*Residues different from the reference are in bold. GII.17, norovirus genogroup II genotype 17; ORF, open reading frame; VP, viral protein.
†First case of norovirus GII.17 Kawasaki in this study (NS-405 from Hong Kong; GenBank accession no. KT326180 [complete genome]).
‡Comprised the competent basal haplotype of VP1. GenBank accession nos.: NS-613 (KU561248; Hong Kong), NS-616 (KU561249; Hong Kong), CAU-192 (KU561252; South Korea), and CAU-267 (KU561254; South Korea).
§Residue speculated to affect viral fitness of norovirus GII.17 Kawasaki in humans.

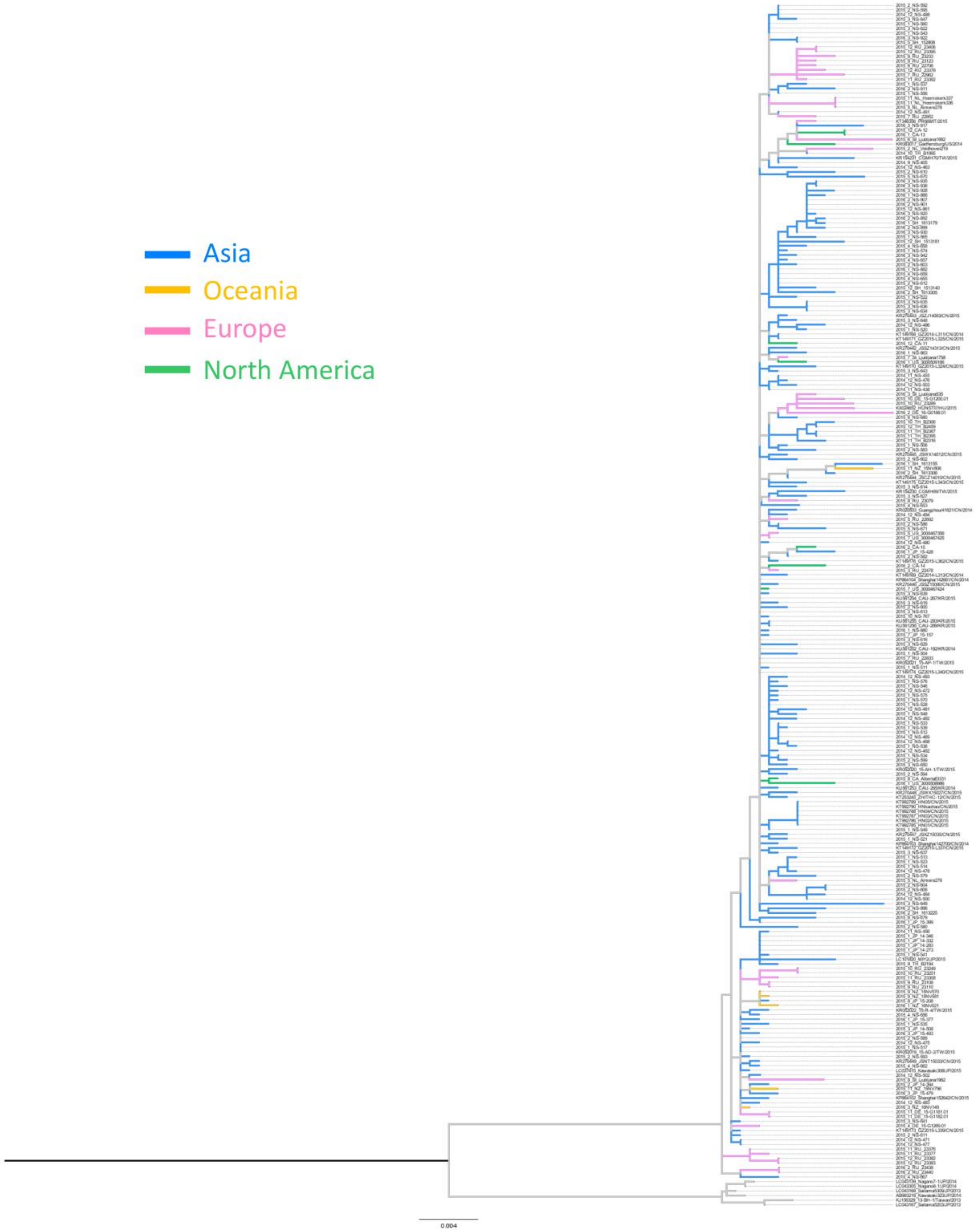


**Technical Appendix Figure.** Maximum-likelihood phylogenetic inference of complete viral protein 1 nucleotide sequences of norovirus genogroup II genotype 17 Kawasaki. This tree is equivalent to that in Figure 1 but with sequence details shown: Year and month of collection, followed by country and strain name. Sequences retrieved from GenBank are preceded with GenBank accession numbers.
